# T194. BRAIN STRUCTURAL AND BEHAVIORAL ABNORMALITIES FOLLOWING PRENATAL EXPOSURE TO MATERNAL INFLAMMATION ARE PREVENTED BY EARLY BUT NOT LATE INTERVENTION

**DOI:** 10.1093/schbul/sby016.470

**Published:** 2018-04-01

**Authors:** Yael Piontkewitz, Michal Arad, Ina Weiner

**Affiliations:** Tel-Aviv University

## Abstract

**Background:**

Prenatal exposure to the viral mimic poly-I:C via the pregnant dam has been shown to lead to a broad spectrum of neuro-psycho-pathological features phenotypic of schizophrenia, recapitulating the well established link between maternal infection in pregnancy and increased schizophrenia risk in the offspring. We previously showed using longitudinal imaging that prenatal poly-I:C leads postnatally to volumetric brain abnormalities that precede the emergence of behavioral abnormalities (selective attention deficits and excessive response to amphetamine) in adulthood, and that an ultra-low dose of risperidone (RIS) given prior to “symptom” emergence, prevents the emergence of behavioral as well as structural brain deficits. Here we aimed at showing that the efficacy of RIS (assessed at adulthood) is a function of time of administration, with earlier but not later being effective.

**Methods:**

On gestation day 15, pregnant Wistar rats were injected IV with polyI:C (4 mg/kg/ml) or saline. Offspring received daily injections of RIS (0.045 mg/kg) at 4 different time windows (TW): PNDs 34–47 (TW1), PNDs 48–61 (TW2), PNDs 62–75 (TW3) or PNDs 106–120 (TW4), and underwent structural imaging (scanned on a 7.0 T/30 cm; Bruker, Rheinstetten, Germany) and behavioral assessment of amphetamine-induced activity (AIA) and latent inhibition (LI). Excessive AIA is considered to mimic the exacerbation of psychotic symptoms in response to amphetamine in schizophrenia patients. LI reflects the normal attentional capacity to ignore stimuli that were experienced as irrelevant in the past; disrupted LI is considered to reflect a failure to ignore irrelevant stimuli and is seen in amphetamine-treated rodents and humans, in schizotypal persons, and in acutely psychotic schizophrenia patients. Testing was conducted on PNDs >90 (for TWs 1–3) and PNDs 106 and 150 (for TW4).

**Results:**

Poly-I:C offspring had larger lateral ventricles (LV) and smaller prefrontal cortex (PFC), striatal (STR) and hippocampal (HIP) volumes, as well as disrupted LI and enhanced AIA compared to their controls. The effects of RIS on these abnormalities was a function of the administration window. RIS was fully effective when given in TW1 (significant prenatal treatment x drug treatment interaction for each of the brain regions as well as for both behavioral indices, and significant differences between poly-IC-vehicle but not between poly-I:C-RIS and the control groups). In TW2, RIS prevented all abnormalities with the exception of HIP volume reduction (statistics as above except for HIP region). In contrast, in both TW 3 and 4, RIS failed to prevent both structural and behavioral abnormalities (only main effect of prenatal treatment for volumetric and AIA measures and only prenatal treatment x preexposure interaction for LI).

**Discussion:**

These results provide the first demonstration that intervention must indeed be provided early in order to be effective, with efficacy declining as the animal matures. Furthermore, they provide strong support for structure-function relationship because there is no prevention of behavioral abnormalities without prevention of structural abnormalities. Finally, since at the 0.045mg/kg dose RIS is a selective 5HT2A/2C antagonist, our results suggest that such antagonism is a promising route for prevention in high risk individuals.

